# Recurrent arterial and venous thrombosis in a 16-year-old boy in the course of primary antiphospholipid syndrome despite treatment with low-molecular-weight heparin: a case report

**DOI:** 10.1186/1752-1947-7-221

**Published:** 2013-08-23

**Authors:** Malgorzata Biernacka-Zielinska, Joanna Lipinska, Joanna Szymanska-Kaluza, Jerzy Stanczyk, Elzbieta Smolewska

**Affiliations:** 1 Department of Pediatric Cardiology and Rheumatology, Medical University of Lodz, 36/50 Sporna St., 91-738, Lodz, Poland

**Keywords:** Antiphospholipid syndrome, Arterial thrombosis, Systemic lupus erythematosus, Venous thrombosis

## Abstract

**Introduction:**

Antiphospholipid syndrome is a multisystem autoimmune disease characterized by arterial and/or venous thrombosis and persistent presence of antiphospholipid antibodies. It can be a primary disease or secondary when associated with other autoimmune diseases.

**Case presentation:**

We present a case of a 16-year-old Caucasian boy with a massive arterial and venous thrombosis in his lower limbs as well as in his central nervous system with clinical symptoms such as headaches and chorea. He did not present any clinical or laboratory signs of a systemic inflammatory connective tissue disease, including systemic lupus erythematosus. Based on the clinical picture and results of the diagnostic tests (positive antibodies against β_2_-glycoprotein and a high titre of anticardiolipin antibodies) we finally diagnosed primary antiphospholipid syndrome. During a 9-month follow up after the acute phase of the disease, he was treated with low-molecular-weight heparin. Neurological symptoms were relieved. Features of recanalization in the vessels of his lower limbs were observed. After a subsequent 6 months, because of the failure of preventive treatment – an incident of thrombosis of the vessels of his testis – treatment was modified and heparin was replaced with warfarin.

**Conclusion:**

Although the preventive treatment with warfarin in our patient has continued for 1 year of follow up without new symptoms, further observation is needed.

## Introduction

Antiphospholipid syndrome (APS), which is also called Hughes syndrome, is characterized by symptoms of thrombosis of the venous, arterial and capillary vessels, and recurrent miscarriages in combination with autoantibodies directed against the proteins that form complexes with phospholipids and participate in the process of blood coagulation. Antibodies against β_2_-glycoprotein (β_2_-GPI), lupus anticoagulant (LAC) or anticardiolipin (aCL) antibodies belong to the class of antiphospholipid (aPL) antibodies. Less frequently, the antibodies can be directed against other antigens such as prothrombin, phosphatidylserine, thrombin, active C protein, S protein, tissue factor XI, plasminogen activator or annexin A5 [1-3]. APS can be primary (pAPS) or secondary (sAPS) when it is associated with other diseases. APS can be associated with systemic diseases of the connective tissue, and particularly with systemic lupus erythematosus, other autoimmune diseases such as diabetes mellitus, thyroid gland inflammation, inflammatory bowel diseases, sarcoidosis, viral infections, tumors, compact tumors as well as lymphoproliferative disorders. The etiology of this syndrome has not been explained and associations of the pathological process with genetic factors (presence of HLA-DR7, DR4, DQw7, DRw53) and infectious factors [1,2,4-7] have been studied. According to the revised classification criteria for APS it can be diagnosed based on more than one clinical and one serological symptom [8].

The antibodies can disturb the results of *in vitro* coagulation tests, such as activated partial thromboplastin time (APTT), kaolin time or prothrombin time, which depend on anionic phospholipids. However, the above antibodies are not detected in approximately 10% to 15% of patients with typical APS symptoms (seronegative APS), which can occur during episodes of vascular occlusion, probably due to binding antibodies in the tissues [8,9]. Clinically, thrombosis most commonly affects the veins of the lower limbs and is often associated with pulmonary thrombosis and hypertension. Arterial thrombosis commonly involves brain vessels, particularly in young people (infarcts, migraine headaches, seizures, disturbances of cognitive functions); it can also involve the coronary arteries or result in renal vein thrombosis. Thrombocytopenia belongs to the most common hematological manifestations of APS [10-17].

## Case presentation

**Figure 1:**
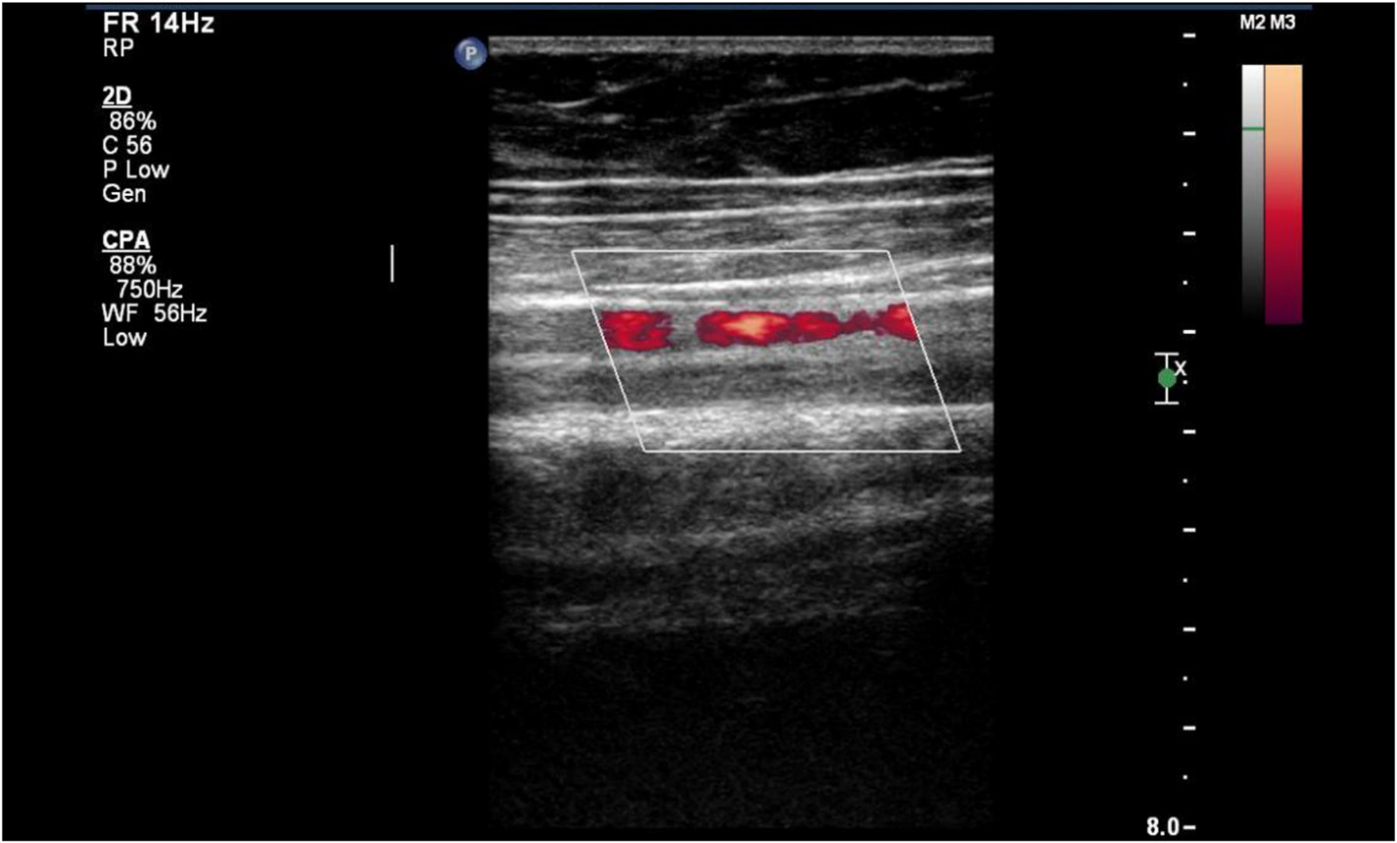
Thrombosis of the left femoral vein.

**Figure 2:**
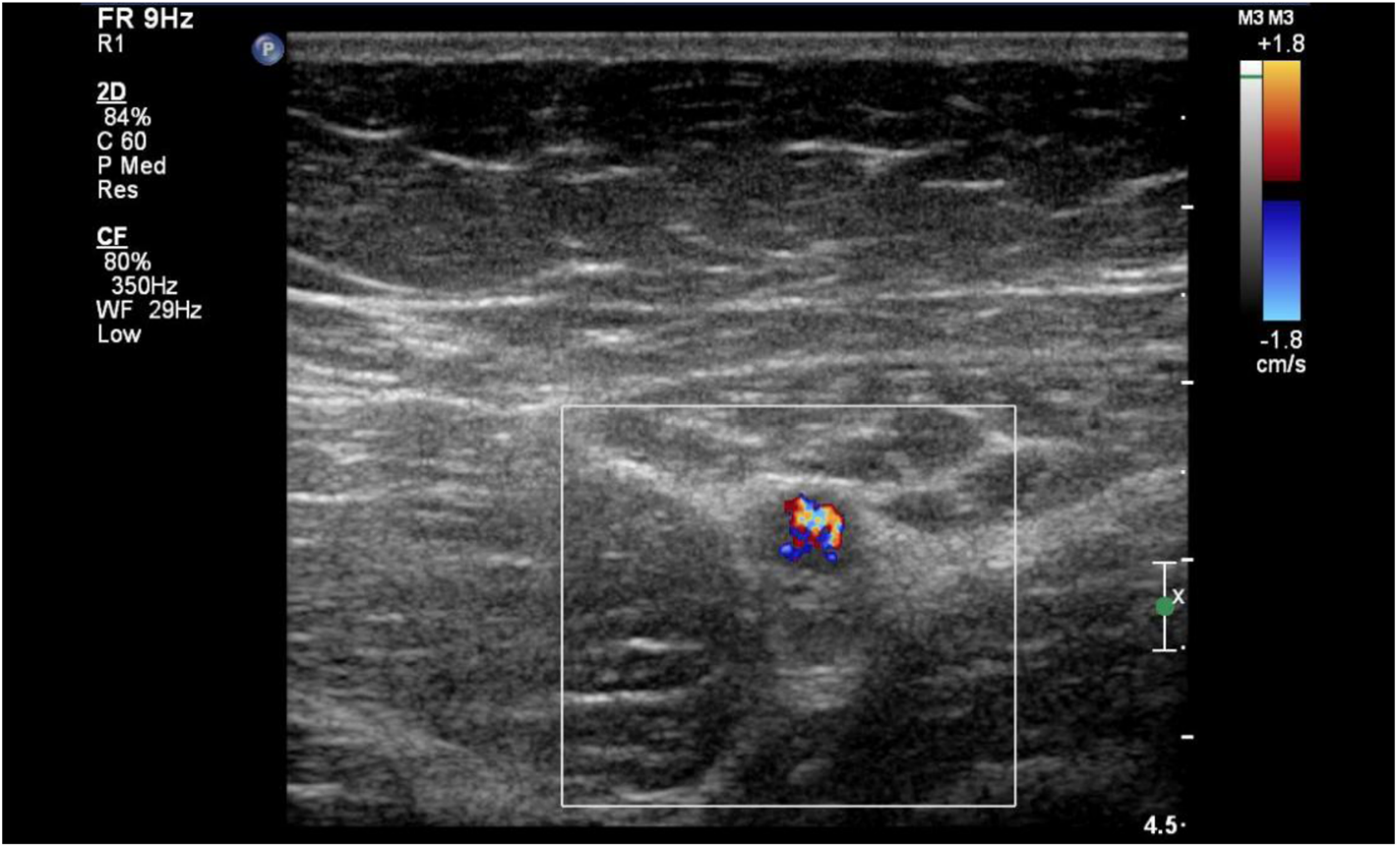
Thrombi in the lumen of the left femoral vein.

A 16-year-old, healthy Caucasian boy, physically active and practicing sports, began suddenly complaining of a sharp pain in his left lower limb, which worsened while walking. The enlarged circumference, reddening and increased temperature of his left lower limb was observed as well. His history revealed that pain in his lower limbs and heels, marked fatigue and limited physical activity occurred as early as 2 weeks before the examination. Initially, he was hospitalized at the Department of Pediatric Surgery in a district hospital and deep vein thrombosis in his femoral vein up to approximately 10cm above the knee joint space was diagnosed. The results of laboratory tests revealed prolonged APTT: 64.7seconds. The remaining coagulation parameters including D-dimers were normal. The patient was administered low-molecular-weight (LMW) heparin (enoxaparin 40mg daily), diosmin, and compression therapy. Following discharge from the district hospital, he was admitted to the Department of Children Surgery and Oncology 12 days later, due to increasing lower limb edema and pain; femoral vein thrombosis in his left lower limb was confirmed. The results of laboratory tests showed markedly prolonged APTT, up to values that could not be determined by laboratory methods, and an increased level of C-reactive protein (Table 1). An intravenous antibiotic was applied (amoxicillin-clavulanate) and the dose of LMW heparin was increased (dalteparin 60mg daily). Compression therapy was continued. However, no clinical improvement was observed in his left lower limb after a month’s therapy; pain in his right foot occurred and his right foot was found to be colder. In addition, no improvement in his left lower limb was observed in a Doppler sonographic examination. On the right side, a two-phase blood flow with slightly weaker pulsation at the level of the adductor canal and the popliteal artery was found in his common femoral artery as well as in superficial and deep femoral arteries. Approximately 12cm to 13cm below the knee joint space, blood flow was growing weaker in his tibial arterial trunks and changed into one-phase flow, with medium pulsation. Peripherally, further deficiency of pulsation was noted in the posterior tibial artery (it was one-phase and characterized by low pulsation); no flow was detected in the right anterior tibial artery. Considering the course of the disease with venal and arterial thrombosis, a suggestion of an autoimmune disease was put forward. The patient was admitted to the Department of Pediatric Neurology 7 days later because of the occurrence of involuntary movements of his upper and lower left limbs as well as the left part of his face. Involuntary movements of the kind of hemilateral chorea involving his upper left limb were observed. A Doppler examination of his lower limbs was performed again and signs of deep vein thrombosis were found. The thrombotic process involved the femoral vein from the level of approximately 7cm below the peripheral ostium of the vena saphena magna (VSM), the popliteal vein and the proximal parts of the 3cm- to 5cm-long left lower limb veins. The lumen of the veins was totally filled with thrombi, which were 10mm to 11mm thick. The thrombi were partially organized. No signs of vessel recanalization were found. Thrombosis was not recognized in the superficial veins. The flow from the limb was via the patent VSM and the iliac vein (Figures 1 and 2).

**Table 1:** Results of laboratory tests performed during the consecutive hospital stays and follow up

**Date of test**	**Hgb g/dL**	**Hct %**	**WBC g/L**	**Platelets mm**^**3**^	**CRP mg/L**	**APTT seconds (N/27–41)**	**PT index % (N/80–120)**	**PT time seconds (N/11–15)**	**D-dimers μg/mL (N<0.5)**	**INR (N/0.8–1.2)**	**Fibrinogen mg/dL (N/200–400)**
February 28 2011	13.8	40.0	9.6	131.000	4.54	**64.7**	94.6	14.1	0.33	-	-
March 10 2011	14.4	43.2	5.6	145.000	**6.1**	Markedly prolonged*	-	14.1	0.33	1.0	312.0
May 16 2011	14.6	41.6	5.1	169.000	2.1	**161.0**	**70.27**	15.7	0.19	1.12	301.27
							**Follow up**				
Jun 22 2012	14.9	43.1	4.8	202.000	0.7	**82.7**	-	**18.2**	**1.21**	**1.76**	332.0

Abbreviations: *APTT* activated partial thromboplastin time, *CRP* C-reactive protein, *Hct* hematocrit, *Hgb* hemoglobin, *INR* international normalized ratio, *N* normal range, *PT* prothrombin, *WBC* white blood cells.

* up to values that could not be determined by laboratory methods.

Both the deep and superficial venous vessels of his right lower limb were permeable and without parietal clots. The vessels were smooth and reacted properly to pressure. The flow in cervical vessels was assessed as well and it was found normal in the common internal and external carotid arteries. The echocardiography revealed normal structure and function of the heart. Due to persistent neurological symptoms computed tomography of the patient’s head was performed. Based on the results, thrombosis of the left sigmoid sinus was suspected.

Magnetic resonance angiography was performed in order to verify the suspected lesions. However, the examination did not confirm thrombosis in the left sigmoid sinus. The suspicion of thrombosis of the internal carotid vein at the level of the skull base was put forward.

The boy was admitted to the Department of Pediatric Cardiology and Rheumatology for the purpose of performing further diagnostic studies. Analysis of the previous diagnostic findings was made and the diagnostic process was broadened according to the suspicion of a systemic inflammatory connective tissue disease.

In order to exclude a systemic connective tissue disease including systemic lupus erythematosus, a number of immunological tests were performed (rheumatoid factor, lupus erythematosus cell test, antinuclear antibodies, anti-neutrophil cytoplasmic antibodies), however, the results were negative. In addition, while searching for the causes of thrombosis, C protein, S protein and homocysteine concentrations were determined and were found to be within the normal range. No mutation of the allele of the gene factor V 1691 G-A (V Leiden) was observed. The patient was GG homozygote (normal sequence). No mutation in the prothrombin gene allele was found either. In both tests his deoxyribonucleic acid was isolated using the commercial kit (CHEMAGEN). The G20210A mutation in the prothrombin gene was detected using the restriction fragment length polymorphism (RFLP) method with the HINDIII enzyme (Fermentas). The mutation in the factor V Leiden was determined using the RFLP method with MNLL enzyme (Fermentas). Because of the fact that thrombosis occurring in the course of the APS was suspected, appropriate diagnostic procedures were performed. The obtained results are presented in Table 2.

**Table 2:** Results of laboratory tests for diagnosing antiphospholipid syndrome

**Date of test**	**LAC**	**aCL IgM (U/mL)**	**aCL IgG (U/mL)**	**β**_**2**_**-GPI IgM (SMU)**	**β**_**2**_**-GPI IgG (SGU)**
March 22 2011	No	**31.14**	**120.0**	Not determined	Not determined
		N<12	N<12		
May 6 2011	No	**25.86**	14.25	14.25	107.40
		N<12	N (0–20)	N (0–20)	N (0–200)
**Follow up**					
June 22 2012	**2.35**	**0.169**	0.072	Not determined	Not determined
	N<1.2	N<0.109	N<0.156		

Abbreviations: *aCL* anticardiolipin, *GPI* glycoprotein, *Ig* immunoglobulin, *LAC* lupus anticoagulant, *N* normal range.

SMU and SGU are the standard units for IgM and IgG antibodies anti- β2GPI (antibodies to β2 glycoprotein) using ELISA method.

Markedly increased levels of aCL and positive results for the anti-β_2_-GPI were noted twice. The patient did not present any sign, except chorea, suggestive of lupus erythematosus or any other systemic inflammatory connective tissue disease. Despite negative LAC, pAPS was diagnosed. He was treated with LMW heparin (dalteparin 15,000IU daily), acetylsalicylic acid at a dose of 75mg daily and risperidone, according to the neurologist’s suggestion. After 9 months of therapy, he remained in a good general condition, and no signs of chorea were observed. However, he complained of insomnia, troubles with memorizing and low mental concentration. Recanalization at the level of the left subpopliteal vein was observed, however, the superficial femoral vein from the level of approximately 6cm of the saphenous vein orifice to the popliteal vein was still filled with clots and no flow was observed. Similarly, a hyperechogenic thrombus was visualized in the distal part of the internal left carotid vein.

He was admitted to the hospital 6 months later due to painful testis edema. Ultrasonography confirmed another episode of venous thrombosis. The treatment was modified and LMW heparin was replaced with warfarin at the alternated dose of 5mg and 7.5mg every second day under international normalized ratio control. After 1.5 years of follow up the therapy with warfarin continues and he has no clinical symptoms.

## Discussion

APS has been considered the most frequent autoimmune thrombotic state in children. Despite a lot of unknown data, it has been quite well described in terms of clinical and laboratory characteristics [2,10,11]. However, there are scarce reports concerning this disease in young patients. Ravelli and Martini analyzed 50 cases of pediatric APS and concluded that the course of the disease in children was similar to adults with chorea and jugular vein thrombosis being recognized more frequently in children, which also occurred in our case [14]. Primary APS is very rare in pediatrics. The onset of the disease before the age of 15 has been estimated to occur in only 2.8% of cases. The occurrence of sAPS in children in the course of systemic lupus erythematosus is more frequent and accounts for 9% to 14% of cases [1-3,7].

The current criteria for adults are also applicable for children but are not fully useful. Obstetric problems, atherosclerosis or the use of contraceptives do not relate to young patients. However, symptoms that are frequent in adults but observable also in children such as *livedo reticularis*, thrombocytopenia and chorea are not included in the criteria [8,10]. One of the indications to extend the immune system evaluation was chorea that occurred in our patient. The Ped-APS Registry, a collaborative project from the European Forum on Antiphospholipid Antibodies and the Juvenile Systemic Lupus Erythematosus Working Group of the Pediatric Rheumatology European Society, gathers data on APS in children [11].

A low level of aPL and anti-β_2_-GPI is found in 7% to 11% of healthy children. A slightly increased level of β_2_-GPI is observed in children with atopic dermatitis and juvenile idiopathic arthritis. A correlation between an increased level of aCL and previous bacterial or viral infections or vaccination has been proven [4]. In the case of our patient, levels of both aCL antibodies and anti-β_2_-GPI were determined twice and found to be significantly increased. Despite negative LAC and normal D-dimers pAPS was diagnosed, and after a year of follow up the patient’s LAC was positive and his D-dimers were elevated (Table 2) [12].

The manifestation of APS in children is similar to adults with primary and secondary syndromes. The main syndrome is thrombosis of veins or arteries. The thrombosis episode may be spontaneous or provoked by an injury, operation, venous stasis, use of contraceptive drugs and so on. In our patient the first incidence of thrombosis occurred after a strenuous march. Despite treatment with heparin, arterial thrombosis in his other limb occurred after a short period of time. LMW heparin is an efficient and safe alternative to standard anticoagulation therapy with unfractionated heparin and oral anticoagulants for both treatment and prevention of thromboembolic events in children of varying ages and underlying disorders. Anticoagulation with heparin followed by oral anticoagulants is a standard treatment in the event of an acute venous or arterial thrombosis. However, in infants and young children resistance to anticoagulation with heparin and vitamin K antagonists, for example warfarin, is sometimes observed, and doses of heparin and LMW heparin higher than those for adults are required to achieve the same anticoagulant effect [2,13].

In children, symptoms concerning inferior and superior vena cava, liver vessels (Budd–Chiari syndrome), heart, kidneys and skin are recognized less frequently than in adults but we did not observe them in our patient.

However, neurological complications such as chorea, migraines, increase of intracranial pressure, psychoses, depression, epilepsy, Guillain–Barré syndrome, transverse damage of the medulla and optic nerve inflammation occur relatively frequently in children. Our patient reported headache, involuntary movements of his face and his lower limb during the formation of thrombus in his jugular vein.

Italian researchers conducted a retrospective study concerning 14 children with pAPS. They found that arterial and venous thrombosis occurred more frequently in children than in adults and in a greater number of males in the studied group. The most frequent common symptom was venous thrombosis of lower limb, profound veins and symptoms from the central nervous system. In four cases the children developed symptoms of systemic lupus or Hodgkin’s lymphoma [14,17].

## Conclusions

The case of pAPS we described in a 16-year-old boy is a relatively rare example of this disease in children and adolescents. However, sAPS is diagnosed more and more frequently. Further observation of vascular lesions and possible new clinical symptoms seems necessary in our patient because of the relatively short course of the disease.

## Consent

Written informed consent was obtained from the patient’s legal guardian for publication of this case report and any accompanying images. A copy of the written consent is available for review by the Editor-in-Chief of this journal.

## Competing interests

The authors declare that they have no conflict of interest.

## Authors’ contributions

All authors analyzed and interpreted the patient data and contributed in writing the manuscript. All authors read and approved the final manuscript.
